# Association of *Neisseria gonorrhoeae* Opa_CEA_ with Dendritic Cells Suppresses Their Ability to Elicit an HIV-1-Specific T Cell Memory Response

**DOI:** 10.1371/journal.pone.0056705

**Published:** 2013-02-12

**Authors:** Qigui Yu, Edith M. C. Chow, Shannon E. McCaw, Ningjie Hu, Daniel Byrd, Tohti Amet, Sishun Hu, Mario A. Ostrowski, Scott D. Gray-Owen

**Affiliations:** 1 Department of Microbiology and Immunology, Indiana University School of Medicine, Indianapolis, Indiana, United States of America; 2 Department of Molecular Genetics, and 3Clinical Sciences Division, University of Toronto, Toronto, Ontario, Canada; University of Pittsburgh, United States of America

## Abstract

Infection with *Neisseria gonorrhoeae (N. gonorrhoeae)* can trigger an intense local inflammatory response at the site of infection, yet there is little specific immune response or development of immune memory. Gonococcal surface epitopes are known to undergo antigenic variation; however, this is unlikely to explain the weak immune response to infection since individuals can be re-infected by the same serotype. Previous studies have demonstrated that the colony opacity-associated (Opa) proteins on the *N. gonorrhoeae* surface can bind human carcinoembryonic antigen-related cellular adhesion molecule 1 (CEACAM1) on CD4^+^ T cells to suppress T cell activation and proliferation. Interesting in this regard, *N. gonorrhoeae* infection is associated with impaired HIV-1 (human immunodeficiency virus type 1)-specific cytotoxic T-lymphocyte (CTL) responses and with transient increases in plasma viremia in HIV-1-infected patients, suggesting that *N. gonorrhoeae* may also subvert immune responses to co-pathogens. Since dendritic cells (DCs) are professional antigen presenting cells (APCs) that play a key role in the induction of an adaptive immune response, we investigated the effects of *N. gonorrhoeae* Opa proteins on human DC activation and function. While morphological changes reminiscent of DC maturation were evident upon *N. gonorrhoeae* infection, we observed a marked downregulation of DC maturation marker CD83 when the gonococci expressing CEACAM1-specific Opa_CEA_, but not other Opa variants. Consistent with a gonococcal-induced defect in maturation, Opa_CEA_ binding to CEACAM1 reduced the DCs’ capacity to stimulate an allogeneic T cell proliferative response. Moreover, Opa_CEA_-expressing *N. gonorrhoeae* showed the potential to impair DC-dependent development of specific adaptive immunity, since infection with Opa_CEA_-positive gonococci suppressed the ability of DCs to stimulate HIV-1-specific memory CTL responses. These results reveal a novel mechanism to explain why infection of *N. gonorrhoeae* fails to trigger an effective specific immune response or develop immune memory, and may affect the potent synergy between gonorrhea and HIV-1 infection.

## Introduction

Gonorrhea, caused by the Gram-negative intracellular diplococcus *Neisseria gonorrhoeae* (*N. gonorrhoeae*), is one of the most prevalent sexually transmitted diseases (STDs) of humans, with over 88 million new cases reported globally each year (http://whqlibdoc.who.int/hq/2011/WHO_RHR_11.14_eng.pdf). *N. gonorrhoeae* infection initiates with the physical attachment of the bacterial surface appendages called pili to the apical side of the host mucosal cells [1], [2]. This loose attachment is followed by a more intimate association involving integral outer membrane protein adhesins, including the colony opacity-associated (Opa) proteins on the bacterial surface [3], [4]. In addition to mediating bacterial attachment, certain Opa variants also promote the transmigration of gonococci across the epithelial layer into the submucosa [4]. A single strain of *N. gonorrhoeae* encodes up to 11 related but antigenically distinct Opa alleles [5]. The expression of each Opa allele is randomly phase-variable and can turn on and off independently [6], [7]. Although a minority of Opa variants can bind to heparan sulfate proteoglycans (HSPG) [7]–[9], the majority of Opa proteins characterized to date target members of the carcinoembryonic antigen-related cellular adhesion molecule (CEACAM) family of receptors [10]–[15], which are expressed not only on various epithelial and endothelial tissues, but also on immune cells [16]–[18].

CEACAMs belong to the immunoglobulin (Ig) superfamily and contribute to the adhesive properties of cells. The human CEACAMs comprise seven members (CEACAM1 and CEACAM3 through CEACAM8) that are characterized by a single amino-terminal Ig variable-like domain and a varying number (0 to 6) of IgG2 constant-like domains [19], [20]. CEACAM1 has the broadest tissue distribution of all characterized CEACAMs and its expression can be induced by neisserial infection or stimulation with a variety of stimuli including 12-O-tetradecanoylphorbol-13-acetate (TPA), calcium ionophore and retinoids [18], [21]–[23]. CEACAM1 has two major isoforms, CEACAM1-L and CEACAM1-S, which differ in their cytoplasmic domains. The cytoplasmic domain of CEACAM1-L consists of 73 amino acids and has 2 immunoreceptor tyrosine-based inhibition motifs (ITIM) that can be phosphorylated to play an inhibitory role through downregulation of intracellular signaling events such as calcium ion influx [24]. CEACAM1-S has a cytoplasmic domain of only 10 amino acids and lacks ITIM motifs [25]. CEACAM1-L and CEACAM1-S are coexpressed at different ratios in different cell types and in different functional states of a cell over time [26].

Despite the availability of effective antibiotic therapies, gonorrhea incidence is rising in the United States and globally after a steady decline for the past two decades [27]–[29]. Although increased screening, use of more sensitive diagnostic tests, and improved reporting may account for a portion of the recent increase, true increases in disease in some populations and geographic areas are also occurring. The success of *N. gonorrhoeae* appears to rely on its ability to avoid the normal development of a memory immune response that would otherwise protect an individual from reinfection during a subsequent exposure. In addition, *N. gonorrhoeae* can subvert immune responses to co-pathogens, such as HIV-1. For example, studies have shown that gonococcal infection is associated with enhanced HIV-1 acquisition and impaired HIV-1-specific CTL responses [30], decreases in blood CD4^+^ T cell count [31], and increased semen and plasma viral loads [31]–[33] in HIV-1 infected individuals, and these are often found to be return to normal with effective gonococcal therapy.

Gonococcal surface epitopes frequently undergo antigenic variation, but this is unlikely to explain the weak immune response to infection as individuals can be re-infected by the same serotype of gonococcal strains [34]–[36]. Recent studies have demonstrated that *N. gonorrhoeae* can directly subvert the natural immune response [37]–[39]. *N. gonorrhoeae* expressing Opa variants that can bind CEACAM1 (herein referred to as Opa_CEA_) were seen to engage this receptor on the surface of primary human CD4^+^ T cells and suppress their activation and proliferation in response to a variety of stimuli [37]. The Opa_CEA_-CEACAM1 binding triggers phosphorylation of CEACAM1 on the tyrosine residuals within the ITIM apparent in the cytoplasmic domain [37], [39]. This allows the recruitment and subsequent activation of the Src homology domain 2 (SH2)-containing tyrosine phosphatases SHP-1 and SHP-2 at the site of bacterial attachment, which prevents the normal tyrosine phosphorylation of the CD3zeta-chain and ZAP-70 kinase in response to T cell receptor (TCR) engagement. This dynamic response allows the bacteria to effectively harness the natural coinhibitory function of CEACAM1 to suppress the adaptive immune response at its earliest step [37]–[39].

Interestingly, CEACAM1 can also inhibit NK cell cytotoxicity when co-ligated with NK cell-activating receptors and the inhibitory effect is mediated by heterophilic interaction between carcinoembryonic antigen (CEA) and CEACAM1 or CEACAM1-CEACAM1 homophilic interaction [40], [41]. Notably, in certain conditions, CEACAM1 is also able to deliver activation signals during an immune response. Triggering CEACAM1 with the monoclonal antibody (mAb) AgB10 can enhance activation and proliferation of murine T cells and B cells, resulting in amplification of immune responses [42]–[44]. In addition, the same mAb (AgB10) induces maturation and chemokine/cytokine secretion of murine dendritic cells (DCs) [25]. It is most likely that the CEACAM1-mAb interaction triggers the activation of these immune cells through activation of the c-Jun NH2-terminal kinase (JNK) pathway [44]. Taken together, these data suggest that CEACAM1 delivers both activation and inhibitory signals, depending on the binding ligand, ratio and activation of the expressed CEACAM1-L and CEACAM1-S isoforms, and the metabolic state of the cell, and that has the capacity to regulate cellular functions of multiple cell types during an immune response.

Dendritic cells (DCs) are professional antigen presenting cells (APCs) that play a critical role in initiating and regulating the adaptive immune response. In recent years, using new cell markers, it has become increasingly apparent that DCs are particularly abundant at mucosal sites and are recruited during infections to the site of mucosal inflammation [45]. They perform a sentinel function for the recognition of invading pathogens and a regulatory function to control mucosal immunity. DCs have been observed to express both long and short forms of CEACAM1, both *in intro* and *in vivo* [25], suggesting that these should be available to bind the neisserial Opa proteins [10], [13], [14]. There is currently no knowledge about the nature of *N. gonorrhoeae* Opa interactions with CEACAM1 on DCs or the functional consequences of these interactions. This study investigates effects of Opa variants on human DC activation and function. We demonstrate that gonococcal expression of Opa­_CEA_ allows bacterial binding to DCs, and downregulates DC maturation in response to infection. Consistent with these effects, DCs infected with Opa­_CEA_-expressing gonococci had a diminished capacity to induce proliferation of allogeneic T cells and were defective in their ability to induce an HIV-1-specific memory CTL response. These effects, when combined with the suppressive effect that Opa-CEACAM1 binding has on primary human T cells, provides an effective strategy by which the bacteria may halt the development of an adaptive immune response.

## Methods and Materials

### Bacterial strains

*N. gonorrhoeae* were grown from frozen stocks on Difco GC agar supplemented with 1% (vol/vol) IsoVitaleX enrichment (BD Biosciences, Mississauga, Ontario, Canada). Isogenic gonococcal strains N302 (Opa-negative, pilus-negative or Opa^-^/P^-^), N303 (constitutively expressing heparan sulfate proteoglycan-specific Opa_50_, herein referred to as Opa_HSPG_), N309 (constitutively expressing CEACAM1 receptor-specific Opa_52_, herein referred to as Opa_CEA_), and N496 (Opa^-^/P^+^) were described previously [14], [46] and were a generous gift from Professor Thomas Meyer (Berlin, Germany). These Opa genes are expressed in a derivative of strain MS11 containing mutations that abolish the expression of the natural chromosomally-encoded HSPG receptor-specific Opa_30_. The ligands recognized by these various Opa variants were previously described [3]. These gonococcal strains were subcultured from frozen stocks and a binocular microscope was used to monitor colony opacity phenotype. Opa expression and variant type were routinely confirmed by SDS-PAGE (10%) and resolved proteins were transferred onto Immobilon P membranes (Millipore, Bedford, MA) and probed with an Opa cross-reactive mAb 4B12/C115 [47]. *Escherichia coli* (*E.coli*) DH5α strain, which was used as a control for gonococcal effects on DC activation and function throughout this study, was obtained from Invitrogen (Carlsbad, CA) and grown on LB agar and LB broth (Sigma-Aldrich, St. Louis, MO).

### Antibodies and reagents

The mouse mAb D14HD11 (IgG1, cross-specific for human CEACAM1, CEACAM3, CEACAM5, and CEACAM6) was a gift of Dr. Fritz Grunert (University of Freiburg, Germany). The murine MOPC-21 IgG1 mAb, used as an isotype control throughout this study, was purchased from Sigma-Aldrich (Sigma-Aldrich, St. Louis, MO). Unless otherwise indicated, anti-human mAbs or polyclonal Abs conjugated with fluorochrome were purchased from BD PharMingen (San Diego, CA): anti-CD3^APC^, anti-CD3^PerCP^, anti-CD8^FITC^, anti-CD14^FITC^, anti-CD1a^FITC^, anti-CD80^FITC^, anti-CD83^PE^, anti-CD86^FITC^, anti-HLA-DR^APC^, anti-IFN-γ^PE^ and anti-IL-12^PE^ (p40/p70), and matched-isotype control Abs conjugated with FITC, PE, PerCP, or APC. Recombinant human granulocyte-macrophage colony stimulating factor (GM-CSF) and interleukin-4 (IL-4) were purchased from ProTech (Rocky Hill, NJ). CD40 ligand trimer (CD40LT) was obtained as a gift from Immunex (Seattle, WA). Cy5-conjugated secondary Abs were obtained from Jackson ImmunoResearch Laboratories (Mississauga, Ontario, Canada). Texas red-X-succinimidyl ester (TRXSE), Bodipy FL-conjugated secondary Abs, and Texas red-conjugated phalloidin were purchased from Invitrogen (Carlsbad, CA).

### Peripheral blood mononuclear cells, monocytes, monocyte-derived DCs, and HIV-1-specific peptides

Peripheral blood mononuclear cells (PBMCs) were isolated by centrifugation on Ficoll-Hypaque (Amersham Pharmacia Biotech AB, Uppsala, Sweden) from heparinized human peripheral blood obtained from healthy blood donors, and frozen at –152°C until use. Monocytes were separated from the PBMCs using multistep Percoll (Sigma-Aldrich, St. Louis, MO) gradient centrifugation, and then purified by depletion of contaminating B cells, T cells, NK cells, and granulocytes using Ab-conjugated magnetic beads in the Monocyte Negative Isolation Kit (Dynal, Oslo, Norway) according to the manufacturer’s guidelines. The resulting cell preparations contained more than 95% monocytes and virtually no residual T and B lymphocytes, as assessed by CD14, CD3 and CD19 staining and flow cytometric analysis. Monocyte-derived DCs (MDDCs) were generated from the purified monocytes by a modification of a method previously described [48], [49]. Briefly, purified monocytes were cultured at 1×10^6^ cells/ml in complete RPMI 1640 medium consisting of RPMI 1640 medium plus 10% fetal bovine serum (FBS), 2 mM glutamine, 25 mM HEPES, and antibiotics in the presence of 50 ng/ml recombinant human GM-CSF and 100 ng/ml recombinant human IL-4 (PeproTech, Rocky Hill, NJ). GM-CSF and IL-4 were added again on days 3 and 5 with the fresh complete RPMI 1640 medium. After 7 days of culture, more than 50% of the cells were CD1a^high^, MHC class II^+^, CD80^low^, and CD14^-^, which represents an immature DC phenotype. The immature MDDCs (iMDDCs) were used for bacterial binding and infection.

Five untreated HIV-1-seropositive individuals (Pt#1 – Pt#5, Table 1) at different stages of disease were recruited for studying gonococcal effects on HIV-1-specific CTL responses. The clinical profiles of these participants are depicted in Table 1. Leukopheresis was performed to obtain large amounts of PBMCs from each of these patients. Prior to the study, individuals were class I HLA-typed and screened for HIV-1-specific CTL by culturing PBMCs with HLA-restricted HIV-1 peptides and detecting IFN-γ-producing CD8^+^ T cells by ELISPOT assay as previously described [48]–[50]. HLA-restricted HIV-1 peptides were purchased from the PeproTech (Rocky Hill, NJ) and dissolved in RPMI 1640 medium or DMSO. The HIV-1 peptides for individual participants Pt#1 to Pt#5 are shown in Table 1. Informed consent was obtained from each of all participants including healthy blood donors and HIV-1-seropositive individuals in accordance with the guideline for conduction of clinical research at the University of Toronto and St. Michael’s Hospital, Canada. All investigational protocols were approved by the University of Toronto and St. Michael’s Hospital institutional review boards.

**Table 1:** Profiles of HIV-1-infected participants.

Participant No.	Stage of Infection	Years of HIV-1-infected	CD4 Count (/mm)	Viral Load (copies/ml)	CD8 Epitopes	ART^a^
Pt#1	LTNP^b^	11	950	<50	B27/gag/KRVVIILGLNK	No
Pt#2	Acute	0.5	560	>500,000	B8/nef/FLKEKGGL	No
Pt#3	Chronic	9	820	158	A*0201/gag/SLYNTVATI	No
Pt#4	LTNP	6	240	100,000	A*0201/gag/SLYNTVATI	No
Pt#5	Acute	0.6	360	332	A*0201/gag/SLYNTVATI	No

a antiretroviral therapy; ^b^long-term nonprogressors

### Analysis of CEACAM expression

To analyze the surface expression of CEACAM1 on monocytes, iMDDCs and mature MDDCs, the cell suspension was first stained with mAb D14HD11 or a non-specific isotype control mAb as primary Ab, followed by goat anti-mouse IgG1^APC^, and then stained with anti-human CD14^FITC^, or HLA-DR^PerCP^ as indicated. HeLa cell lines transfected with either a human CEACAM1 cDNA-encoding plasmid or the empty pRC/CMV expression vector were used as positive and negative controls for CEACAM1 surface expression, respectively [51]. Peripheral B lymphocytes (CD19^+^) in the PBMCs from each individual were also stained for CEACAM1 expression as these cells are known to display high CEACAM1 surface expression, which could be used for the comparison of CEACAM1 expression on the surface of iMDDCs or mature MDDCs. A human B cell line C1R (a kind gift from Dr. MacDonald K.S. at the University of Toronto) was also used as a positive control for CEACAM1 expression analysis. Stained cells were subjected to flow cytometric analysis to examine surface expression of CEACAM1.

Western blot analysis of CEACAM1 expression was performed as described in our previous report [52]. Briefly, cells were washed once with ice-cold phosphate-buffered saline (PBS) prior to their resuspension in 100 µl of cell lysis buffer (Cell Signaling Technology, Pickering, Ontario, Canada) containing 20 mM Tris-HCl (pH 7.5), 150 mM NaCl, 1 mM Na_2_EDTA, 1 mM EGTA, 1% Triton, 2.5 mM sodium pyrophosphate, 1 mM β-glycerophosphate, 1 mM Na_3_VO_4_, 1 µg/ml leupeptin, and 1 mM phenylmethylsulfonyl fluoride. Cells were incubated for 15 min at 4 °C on an orbital shaker and then centrifuged to pellet the cellular debris. The protein-containing supernatant was stored at -80 °C until use. Equal amounts (15 µl per lane) of the protein-containing supernatants were mixed with 5 µl of 4 X NuPAGE LDS Sample Buffer (Invitrogen, Carlsbad, CA), boiled for 5 min, subjected to NuPAGE Novex high performance gel electrophoresis (Invitrogen, Carlsbad, CA), and then blotted onto polyvinylidene difluoride membranes (Millipore, Billerica, MA). Membranes were blocked with 5% nonfat dry milk in TBS-T buffer (50 mM Tris-HCl, 150 mM NaCl, 0.05% Tween 20) for 1 h at room temperature (RT) and then incubated at 4 °C overnight with CEACAM-specific mAb D14HD11 as a primary Ab. These blots were detected using horseradish peroxidase (HRP)-conjugated secondary Ab (Southern Biotechnology Associates, Birmingham, AL) and visualized using the ECL detection system (Pierce, Rockford, IL). These blots were reprobed with β-actin Abs (Abcam, Cambridge, MA) as loading controls.

### Bacterial binding, internalization and infection

Binding of isogenic gonococcal strains of N302, N303, N309 and N496 to iMDDCs was evaluated using FITC-labeled bacteria. For comparison, FITC-labeled *E.coli* DH5α was also studied side-by-side for bacterial binding. Bacteria (10^9^/ml) were labeled by incubation of 0.5 mg FITC (Sigma-Aldrich, St. Louis, MO) per ml in phosphate-buffered saline (PBS, pH 7.4) at RT for 1 h. The FITC-pulsed bacteria were washed five times with PBS to remove unbound FITC. To test bacterial binding, iMDDCs were incubated with FITC-labeled bacteria at a multiplicity of infection (MOI) of 10 for 30 min at RT. Bacterial binding was determined by measuring the percentage of cells that bound FITC-labeled bacteria using flow cytometric analysis.

To test whether bacterial binding to iMDDCs is mediated by DC-SIGN on the cell surface, mannan blocking assays were performed site-by-site with the bacterial binding tests. Prior to adding FITC-labeled bacteria, iMDDCs were treated with mannan at 5 - 20 µg/ml, the concentrations that have been shown to completely block DC-SIGN-mediated binding of *mycobacterium tuberculosis* to DCs [53], [54], followed by incubation with FITC-labeled bacteria at an MOI of 10 for 30 min at RT. Blockage of bacterial binding was determined by measuring the percentage of bacterial binding deduction (% of cells that bound FITC-labeled bacteria in the absence of mannan minus % of cells that bound FITC-labeled bacteria in the presence of mannan).

Internalization of bacteria by iMDDCs was also investigated. Immature MDDCs were allowed to adhere onto coverslips pre-coated with 0.2% gelatin (Sigma-Aldrich, St. Louis, MO). These cells were pulsed with gonococcal strains (MOI = 100) prelabeled with Texas red-X-succinimidyl ester (Invitrogen, Carlsbad, CA) at 37°C for 1 h. Cells were immersed for 30 min at 37°C in 3.5% paraformaldehyde solution. Extracellular bacteria were then labeled with the polyclonal anti-gonococcal serum (UTR01), which was raised against N. gonorrhoeae N302 (Opa^-^), followed by a staining with a BODIPY-FL-conjugated secondary Ab (Invitrogen, Carlsbad, CA). Immature MDDCs were then permeabilized with 0.4% Triton X-100 and stained with Phalloidin-FITC (Sigma-Aldrich, St. Louis, MO) for visualizing cellular actin filaments. Intracellular (red) versus extracellular (yellow) bacteria with iMDDCs (green) were then distinguished by visualization with a Leica DM-IRBE inverted fluorescence microscope (Leica Microsystems, Toronto, Ontario).

### Induction of HIV-1 peptide-specific CTL

The protocol for expanding circulating memory HIV-1-specific CTL *ex vivo* was described previously [48]–[50]. Immature MDDCs were infected with individual isogenic gonococcal strain or *E.coli* DH5α at 0-100 MOI in complete RPMI 1640 medium with an addition of 1 U/ml of endonuclease (Sigma-Aldrich, St. Louis, MO), which was used to prevent gonococcal aggregation mediated by DNA released through bacterial autolysis. Gentamycin was added at a final concentration of 50 µg/ml (Bishop, Burlington, Ontario) to each condition 6 h after the start of infection and maintained throughout the experimental time-course to prevent gonococcal overgrowth during the 2–3 days of cell culture. Infected cells were subjected to centrifugation on Ficoll-Hypaque (Amersham Pharmacia Biotech AB, Uppsala, Sweden) to remove cellular and bacterial debris prior to co-culture with autologous T cells. In a parallel experiment used as a positive control, iMDDCs were incubated with CD40LT at 1 µg/ml for 2–3 days, which has been shown to significantly expand HIV-1 specific CTLs [48], [49], [55]. Cells from each condition were pulsed with the HIV-1-specific HLA class I-restricted peptide at 40 µg/ml for 1 h at 37°C, then plated in 24-well plates (5×10^5^ pulsed or nonpulsed MDDCs/well) in complete RPMI 1640 medium. Freshly isolated or thawed autologous PBMCs were prepared and then added to the MDDCs at a 1:10 ratio (5×10^6^cells/well in 2 ml medium). The following conditions were included in all experiments: 1) MDDCs not pulsed with peptides; 2) MDDCs pulsed with peptides; and 3) MDDCs infected with N302, N303, N309, N496, or *E.coli* DH5α, or treated with CD40LT, then pulsed with peptides. On days 3, 5, and 7, the medium was changed. On day 10, duplicate wells were pooled and cells were harvested and tested for HIV-1-specific CTL activity by intracellular IFN-γ staining. Experiments were repeated in Pt#1 and Pt#2 samples.

### Flow cytometric analysis

Surface staining of PBMCs, isolated monocytes, and immature or mature MDDCs were performed in PBS/1% FBS/0.02% NaN_3_ using fluorochrome-conjugated Abs. Events were acquired using FACSCalibur System (BD Biosciences, San Jose, CA) and data were analyzed using FlowJo software (Tree Star Inc., San Carlos, CA). For intracellular staining, cells were permeabilized using reagents in the Cytofix/Cytoperm kit (BD PharMingen, San Diego, CA) in accordance with the manufacturer’s recommendations. Intracellular staining was performed to enumerate the number of IL-12-producing MDDCs or the number of IFN-γ-producing CD8^+^ T cells, as previously described [48], [49], [55]. For IFN-γ-producing CD8^+^ T cell staining, 0.25×10^6^ cells were cultured in U-bottom 96-well plates in the presence of peptide-pulsed (1-10 µM) autologous B-lymphoblastoid cell lines (B-LCL) or autologous T cell-depleted PBMCs as stimulator cells; nonpeptide-pulsed stimulator cells were used as background controls. Positive control cells were stimulated with the bacterial superantigen staphylococcal enterotoxin B (SEB, 1 µg/ml) (Sigma-Aldrich, St. Louis, MO). Cells were incubated with peptide-pulsed or nonpeptide-pulsed stimulator cells for 6 h at 37°C in 5% CO_2_. Monensin was added for the duration of the culture period to facilitate intracellular cytokine accumulation. A total of 50,000–100,000 events were collected from each sample for intracellular IFN-γ assay, and CD8^+^ T cells were enumerated after gating on CD3-positive cells.

### Statistical analysis

Data were compared using the Wilcoxon signed rank test for paired samples. Statistical significant was defined by *p<* 0.05.

## Results

### Expression of CEACAM1 by immature and mature MDDCs

**Figure 1:**
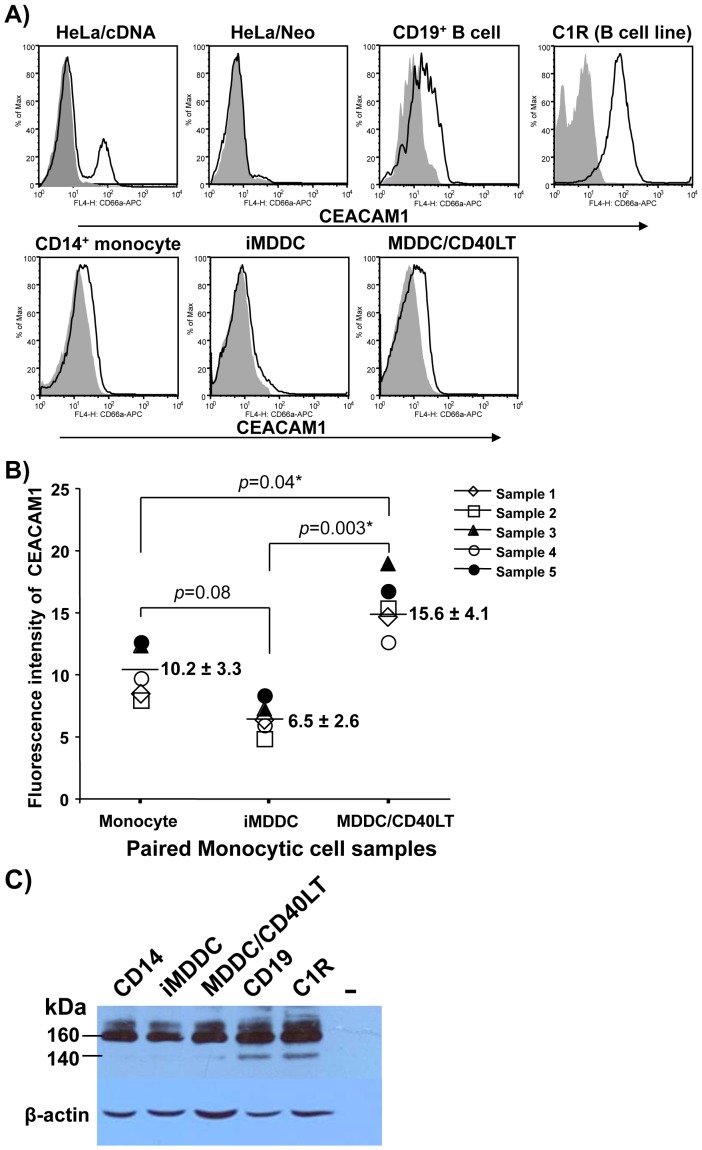
Expression of CEACAM1 by immature and mature MDDCs. ***A)*** The histograms show the log fluorescence intensity of CEACAM1 on the surface of immature and mature MDDCs labeled with either mouse anti-human CEACAM1-speciifc mAb D14HD11 (bold line profiles), or an isotype-matched control mAb (solid gray profiles), followed by fluorochrome-conjugated goat-anti-mouse secondary Ab. HeLa cells transfected with CEACAM1 cDNA (HeLa/cDNA) or empty plasmid vector (HeLa/Neo) were used as positive and negative staining controls of CEACAM1 expression, respectively. Peripheral CD14^+^ monocytes, CD19^+^ B cells, and C1R cells were also included for comparison of surface expression of CEACAM1 and expression phenotypes. The data show one representative staining from five healthy blood donors. ***B)*** Summarized data of CEACAM1 expression determined by flow cytometric analysis from five healthy blood donors. Fluorescence intensity was obtained from CEACAM-1 specific D14HD11 mAb staining subtracted from isotype-matched control mAb staining. The number shows the average fluorescence intensity. ***C)*** Western blot analysis of CEACAM1 protein expression. Equal amounts of protein extracts from peripheral CD19^+^ B cells, C1R cells, purified peripheral CD14^+^ monocytes, iMDDCs (harvested from day 7 cultures), and CD40LT-matured MDDCs were subjected to Western blot analysis using D14HD11 as a primary mAb and a secondary Ab labeled with HRP. The blot was reprobed with β-actin Ab as loading controls (lower panel). Data are representative for three independent experiments.

To determine whether CEACAM1 expression is affected by DC maturation, we generated MDDCs from *ex vivo* isolated monocytes by supplementing the primary cultures with recombinant human GM-CSF and IL-4. After 7 days, more than 50% of the cells were CD1a^high^ and CD14^-^, and most cells expressed low or undetectable level of MHC-II molecule HLA-DR and costimulatory molecules CD40, CD80, CD83, and CD86, which together reflects an immature MDDC (iMDDC) phenotype. This population was characterized for CEACAM1 expression, bacterial binding and infection. Flow cytometric analysis using mAb D14HD11, which detects the extracellular domain of human CEACAM1, showed that iMDDCs expressed low, but detectable levels of CEACAM1 on the cell surface (Fig. 1A), which is consistent with the previous observation that mouse DCs express CEACAM1 [25]. CEACAM1 expression on iMMDCs was slightly, but not significantly, down-regulated when compared with the monocytes (Fig. 1A, 1B). The expression of this protein was significantly up-regulated (2- to 3-fold) when the iMDDCs were subcultured in the presence of soluble recombinant human CD40LT at 1 µg/ml for additional 3 days (Fig. 1B), a condition that drives iMDDC maturation. CEACAM1 expression level on monocytes, iMDDCs, or mature MDDCs was lower than that of peripheral CD19^+^ B cells and C1R cells, which are known to display high CEACAM1 surface expression (Fig. 1A). Different from CEACAM1 cDNA transfected HeLa cells and the C1R cell line, which displayed a clear-cut single CEACAM1-positive peaks (Fig. 1A), primary monocytes, iMDDCs, matured MDDCs and B cells each displayed a gradient of CEACAM1-positive staining on the mass of these cells, indicating that the majority or all cells expressed CEACAM1, but at varying levels.

The expression of CEACAM1 by monocytes, immature or mature MDDCs was confirmed with a Western blot. Monocytes, iMDDCs from day 7 cultures and MDDCs matured by treatment of CD40LT expressed both CEACAM1 isoforms (Fig. 1C). The presence of the L isoform indicated that CEACAM1 expressed on the surface of MDDCs could potentially deliver inhibitory signals via its ITIM motifs [37]. CEACAM1 is thus expressed on the surface of iMDDCs developed *in vitro* from monocytes, and is up-regulated to a limited extent on mature MDDC during their *in vitro* differentiation.

### Contribution of Opa proteins and pili to N. gonorrhoeae interactions with iMDDCs

**Figure 2:**
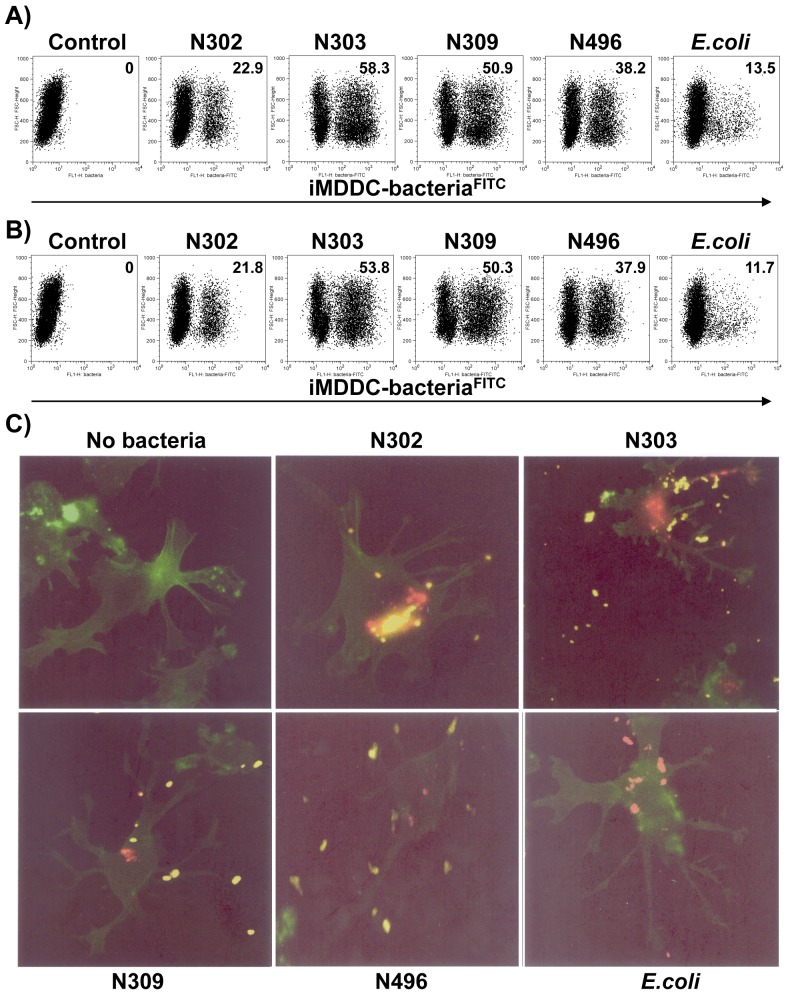
Gonococcal binding and internalization to iMDDCs. ***A*** *)* Immature MDDCs were incubated with FITC-labeled gonococcal strains N302, N303, N309, and N496, or *E.coli* DH5α bacteria at an MOI of 10 for 30 min at RT. Bacterial binding was determined by measuring the percentage of cells that bound FITC-labeled bacteria using flow cytometric analysis. The percentage of cells with bound FITC-labeled bacteria is indicated in each condition. ***B)*** Bacterial binding to iMDDCs is not mediated by DC-SIGN on the cell surface. Mannan blocking assays were carried out site-by-site with the bacterial binding tests. The percentage of cells with bound FITC-labeled bacteria is indicated in each condition in the presence of mannan at 20 µg/ml. ***C)*** Internalization of gonococcal strains and *E.coli* DH5α into iMDDCs. Immature MDDCs were allowed to adhere onto coverslips pre-coated with 0.2% gelatin. These cells were pulsed with gonococcal strains or *E.coli* DH5α (MOI = 100) prelabeled with Texas red-X-succinimidyl ester at 37^o^C for 1 h. Extracellular bacteria were then labeled with the polyclonal anti-gonococcal serum, followed by a staining with a BODIPY-FL-conjugated secondary Ab. Immature MDDCs were then permeabilized with 0.4% Triton X-100 and stained with Phalloidin-FITC. Intracellular (red) versus extracellular (yellow) bacteria with MDDCs (green) were then distinguished by visualization with a Leica DM-IRBE inverted fluorescence microscope. The magnification for all conditions is 40.

In order to understand whether the gonococcal Opa protein adhesins and/or pili influenced association of *N. gonorrhoeae* with iMDDCs, isogenic strains of *N. gonorrhoeae* constitutively expressing defined adhesins were employed. Immature MDDCs were incubated with FITC-labeled isogenic gonococcal strains or *E. coli* DH5α at an MOI of 10 bacteria per cell at RT for 30 min. After fixation with 1% formaldehyde, bacterial binding to cells was determined by flow cytometric analysis to measure the percentage of cells with bound FITC-labeled bacteria. The iMDDCs efficiently bound *N. gonorrhoeae* regardless of Opa expression (Fig. 2A), which was consistent with a previous report [56]. Of the four isogenic gonococcal strains examined, the Opa^-^/P^-^ N302 showed the lowest binding to iMDDCs (20.6±3.6%, n = 3), while N303 (expressing Opa_HSPG_), N309 (expressing CEACAM1 receptor-specific Opa_CEA_), and N496 (Opa^-^/P^+^) all bound to iMDDCs at high levels, with 53.7±6.9% (n = 3), 51.2±7.3% (n = 3), 47.6±7.8% (n = 3), respectively. Compared with *N. gonorrhoeae, E.coli* showed substantially less binding to iMDDCs (11.7±3.2 %, n = 3). Combined, the data indicate that gonococcal binding to iMDDCs did not require an interaction between Opa_CEA_ and CEACAM1, as gonococci lacking Opa_CEA_ effectively adhered to the cells. However, CEACAM1 binding could facilitate bacterial association with the iMDDCs since Opa_CEA_-expressing N309 adhered to a greater extent than did the isogenic Opa-deficient N302. When considered together with previous reports [56], [57], these observations suggest that neither binding nor engulfment of *N. gonorrhoeae* by DCs was not affected by Opa protein or pilus expression.

Considering that binding occurred without Opa or pilus adhesin expression, we investigated whether *N. gonorrhoeae* or *E. coli* bound to iMDDCs through the surface molecule DC-SIGN (dendritic cell-specific intercellular adhesion molecule-3-grabbing non-integrin), which structurally contains a mannan-binding lectin domain [58], [59]. DC-SIGN recognizes a large array of pathogens, including HIV-1 [60], Ebola [61], hepatitis C virus (HCV) [62], [63], Dengue virus [64], *Leishmania amastigotes* [*65*], *Mycobacterium tuberculosis* [53], [54], *Candida albicans* [66], and *Aspergillus fumigatus* conidia [67], in a mannan-dependent manner. For these pathogens, DC-SIGN relies on the presence of mannose residues on surface oligosaccharide chains. More pertinent for this study, certain lipo-oligosaccharide mutants of *N. gonorrhoeae* that expose otherwise internal mannose residues are recognized by DC-SIGN [57]. In each case, soluble mannan at 5 - 20 µg/ml will, therefore, block the binding of these pathogens to DCs [53], [54]. We found that bacterial binding to iMDDCs was not affected by 20 µg/ml mannan treatment, as the percentage of cells that bound FITC-labeled bacteria did not significantly decrease. This indicates that gonococcal binding by iMDDCs was not dependent on DC-SIGN for any strain tested (Fig. 2B).

The high efficiency bacterial binding to iMDDCs prompted us to investigate whether the iMDDC-bound bacteria could be internalized by iMDDCs. Immature MDDCs were pulsed with isogenic gonococcal strains or *E.coli* DH5α that had been prelabeled with Texas red-X, succinimidyl ester (Invitrogen, Carlsbad, CA). Prior to permeabilizing the DC membrane, extracellular gonococcal bacteria were labeled with the polyclonal anti-gonococcal serum (UTR01), which was then detected using a BODIPY-FL-conjugated secondary Ab (Invitrogen, Carlsbad, CA). Intracellular (red) versus extracellular (yellow) gonococci associated with iMDDCs (green) were then visualized by fluorescence microscopy. Both the gonococcal strains and *E.coli* DH5α could be internalized by iMDDCs (Fig. 2C), and this activity was not blocked by mannan (data not shown). In all four isogenic gonococcal strains tested, both extracellular (yellow) and intracellular (red alone) bacteria could be observed, indicating that the bound bacteria could be internalized. In contrast, only bacteria (red) labeled with Texas red-X, succinimidyl ester were visualized for *E. coli* DH5α, confirming the specificity of extracellular staining (yellow) with anti-*N. gonorrhoeae* N302 polyclonal Abs (Fig. 2C).

### Association of N. gonorrhoeae Opa_CEA_ with MDDCs down-regulates CD83 expression

**Figure 3:**
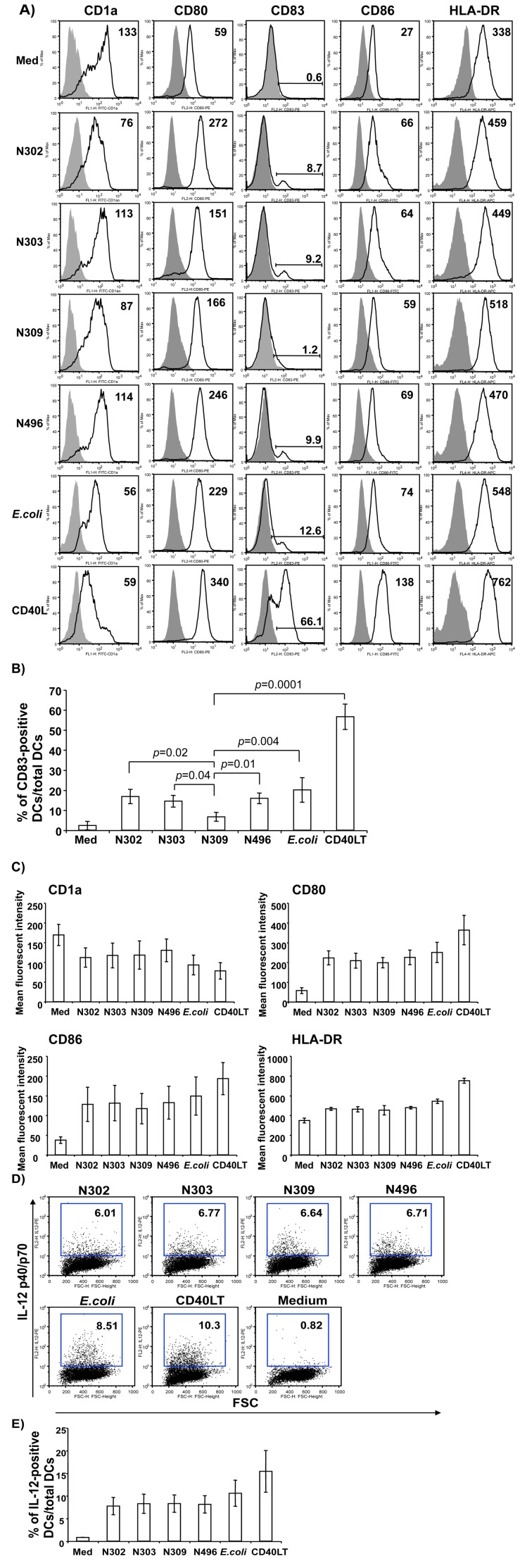
Association of *N. gonorrhoeae* Opa_CEA_ with MDDCs down-regulated CD83 expression, but did not affect IL-12 induction. ***A)*** A representative experiment from a healthy participant is shown. Immature MDDCs were infected with individual isogenic gonococcal strain or *E.coli* DH5α at an MOI of 10 in complete RPMI 1640 medium. Medium alone and CD40LT were included for negative and positive controls, respectively. MDDCs were harvested and expression of surface molecules was assayed by flow cytometric analysis. Values represent the mean flurorescence intensity subtracted from the value of matched isotype control mouse mAbs (shaded gray histogram). ***B)*** Summary data of CD83 expression on MDDCs surface obtained from all five participants of healthy blood donors are shown. Statistical comparisons of data pooled from five participants were performed between Opa_CEA_-expressing N309 and other gonococci strains, *E.coli* DH5α, or CD40LT treatment: N309 vs N302, *p* < 0.05; N309 vs N303, *p* < 0.05; N309 vs N496, *p* < 0.01; N309 vs *E.coli*, *p* < 0.005; N309 vs CD40LT, *p* < 0.0001; CD40LT  =  CD40 ligand trimer. ***C)*** Summary data on expression of CD1a, CD80, CD86, and HLA-DR on MDDCs surface obtained from all five participants of healthy blood donors are shown. ***D)*** A representative experiment is illustrated to show the effects of *N. gonorrhoeae* infection on IL-12 induction by MDDCs. Immature MDDCs were infected with Opa_CEA_-positive *N. gonorrhoeae* N309, or Opa_CEA_-negative gonococcal strains N302, N303, N496 or *E.coli* DH5α, or treated with CD40LT at 1 µg/ml for 12 h, and IL-12 induction was measured by intracellular staining and flow cytometric analysis. The numbers (inset upper right) represent the percentage of IL-12 p40/p70-positive MDDCs per total MDDCs. Data are taken from one of HIV-1-uninfected participants and are representative of experiments with MDDCs derived from HIV-1-infected and HIV-1-uninfected individuals. ***E)*** Pooled data from all participants are shown.

Immature DCs are highly efficient in antigen capture and processing, whereas mature DCs are specialized in antigen presentation and activation of naive T cells to evoke cellular immune responses. Immature DCs mature in response to various signals, including bacterial components (lipopolysaccharide or LPS), inflammatory cytokines (TNF-α, PGE2) or co-stimulatory molecules (CD40L). We investigated whether *N. gonorrhoeae* affected the normal maturation of MDDCs in response to infection. Relative to medium alone, all bacterial infections induced marked changes in the cellular morphology of the iMDDCs, consistent with those occurring in response to the potent inducer (CD40-ligation) of DC maturation (data not shown). The bacterial infections generally also led to changes in expression of surface molecules known to correlate with maturation. As shown in Fig. 3A, the MDDCs cultured with medium alone were HLA-DR^low^, expressed high levels of CD1a, and low or undetectable levels of CD80, CD83, and CD86, typical of an immature DC phenotype. Among the bacterial strains, *E. coli* DH5α was the most potent inducer of DC maturation, as demonstrated by decreased CD1a expression and increased CD80, CD83, and CD86 expression (Fig. 3A, 3B). *N. gonorrhoeae* infection tended to induce similar changes in MDDC maturation markers regardless of adhesin expression, with the exception that CD83 was unexpectedly down-regulated in the samples infected with Opa_CEA_-positive gonococcal strain N309 (6.7 ± 2.2, n = 5). In contrast, the Opa-negative gonococcal strain N302 (16.8 ± 3.6, n = 5), Opa_HSPG_-expressing N303 (14.5 ± 2.8, n = 5), and piliated N496 (15.9 ± 2.7, n = 5) all induced CD83 levels similar to that of the *E. coli* control, indicating that interaction of Opa_CEA_ with CEACAM1 affected MDDC maturation by suppressing CD83 (Fig. 3A, B). It is pertinent to note that, other than CD83, there were no other apparent differences between N309, the other gonococcal strains or *E.* coli with regards to its effect on CD1a, HLA-DR, CD80, and CD86 (Fig. 3A, 3B, 3C), suggesting that this was a CD83 specific effect.

IL-12 is a pleiotropic cytokine that is secreted by activated professional APCs, including DCs. IL-12 can induce Th1-type cellular responses, T cell proliferation, and IFN-γ secretion from activated T cells and NK cells. We and others previously have demonstrated that MDDCs could be induced to produce IL-12 after CD40 ligation [48], [49], [55], [68]. We thus investigated whether Opa_CEA_ could affect IL-12 production from *N. gonorrhoeae*-infected MDDCs. Immature MDDCs were infected with Opa_CEA_-positive *N. gonorrhoeae* N309, or Opa_CEA_-negative gonococcal strain N302, N303, N496 or *E.coli* DH5α for 12 h, and IL-12 induction was measured by intracellular staining using flow cytometric analysis. A representative experiment is illustrated in Fig. 3D and summary data from all experiments is shown in Fig. 3E. In comparison with medium alone, all isogenic gonococcal strains, regardless of Opa_CEA_ expression, induced IL-12 production by MDDCs, albeit at somewhat lower levels than did *E. coli* DH5α or CD40LT. No significant difference between the levels of IL-12 expression was observed among the isogenic gonococcal strains.

### Association of N. gonorrhoeae Opa_CEA_ with MDDCs decreases the sensitization of allogeneic mixed lymphocyte proliferation

**Figure 4:**
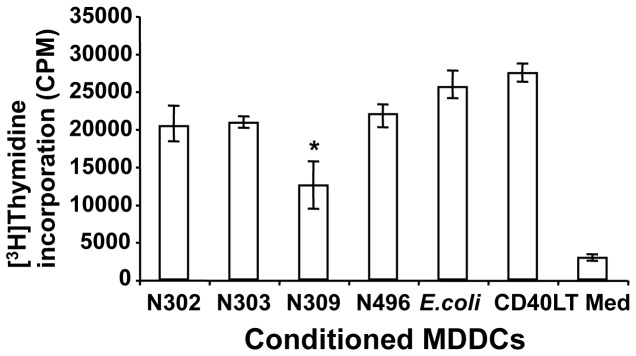
Association of *N. gonorrhoeae* Opa_CEA_ with MDDCs decreased the sensitization of allogeneic mixed lymphocyte proliferations. Immature MDDCs infected, uninfected, or CD40LT-treated for 3 days were plated into 96-well round-bottom tissue-culture plates with allogeneic PBMCs at a ratio of 1:20 (MDDCs to T cells). Cells were cocultured for 3 days and pulsed with 2 µCi (0.076 Mbq) [^3^H]thymidine for an additional 6 h. [^3^H]Thymidine incorporation in harvested cells was assayed by liquid scintillation spectrometry. These data are mean ± SEM of triplicates and representative of four independent experiments with different donors. Statistical comparisons of data pooled from the five participants were performed between Opa_CEA_-expressing N309 and other gonococci strains, *E.coli* DH5α, or CD40LT treatment: N309 vs N302, *p* < 0.05; N309 vs N303, *p* < 0.05; N309 vs N496, *p* < 0.05; N309 vs *E.coli*, *p* < 0.05; N309 vs CD40LT, *p* < 0.001; CD40LT  =  CD40 ligand trimer.

Activated DCs are potent APCs that sensitize T lymphocytes to both allotypic and other antigens. Considering the role of CD83 in T cell stimulation [69], we sought to test whether Opa_CEA_ expression affected the functional capacity of MDDCs that were matured in response to infection with the various *N. gonorrhoeae* strains by measuring induced T lymphocyte proliferation in an allogeneic mixed lymphocyte reaction (MLR). Medium alone and CD40LT were included as negative and positive controls, respectively. As reported previously [48], [49], MDDCs that were matured in response to CD40LT strongly enhanced allogeneic T cell proliferation, measured by thymidine incorporation, when compared with medium alone (Fig. 4). MDDCs that were matured in response to most bacterial infections induced T cell proliferation to a similar degree. *E.coli* DH5α-infected MDDCs were the most potent inducers of T cell proliferation, however Opa_CEA_-negative strains, including N302, N303 and N496, also induced strong T cell proliferation. Unexpectedly when considering that they appeared to be mature by most measures, the MDDCs that had been exposed to the Opa_CEA_-positive strain N309 showed markedly lower T cell proliferation, with the difference being both highly reproducible among donor samples and statistically significant when all were combined (Fig. 4).

### Effects of N. gonorrhoeae infection on HIV-1-specific CTL memory response

**Figure 5:**
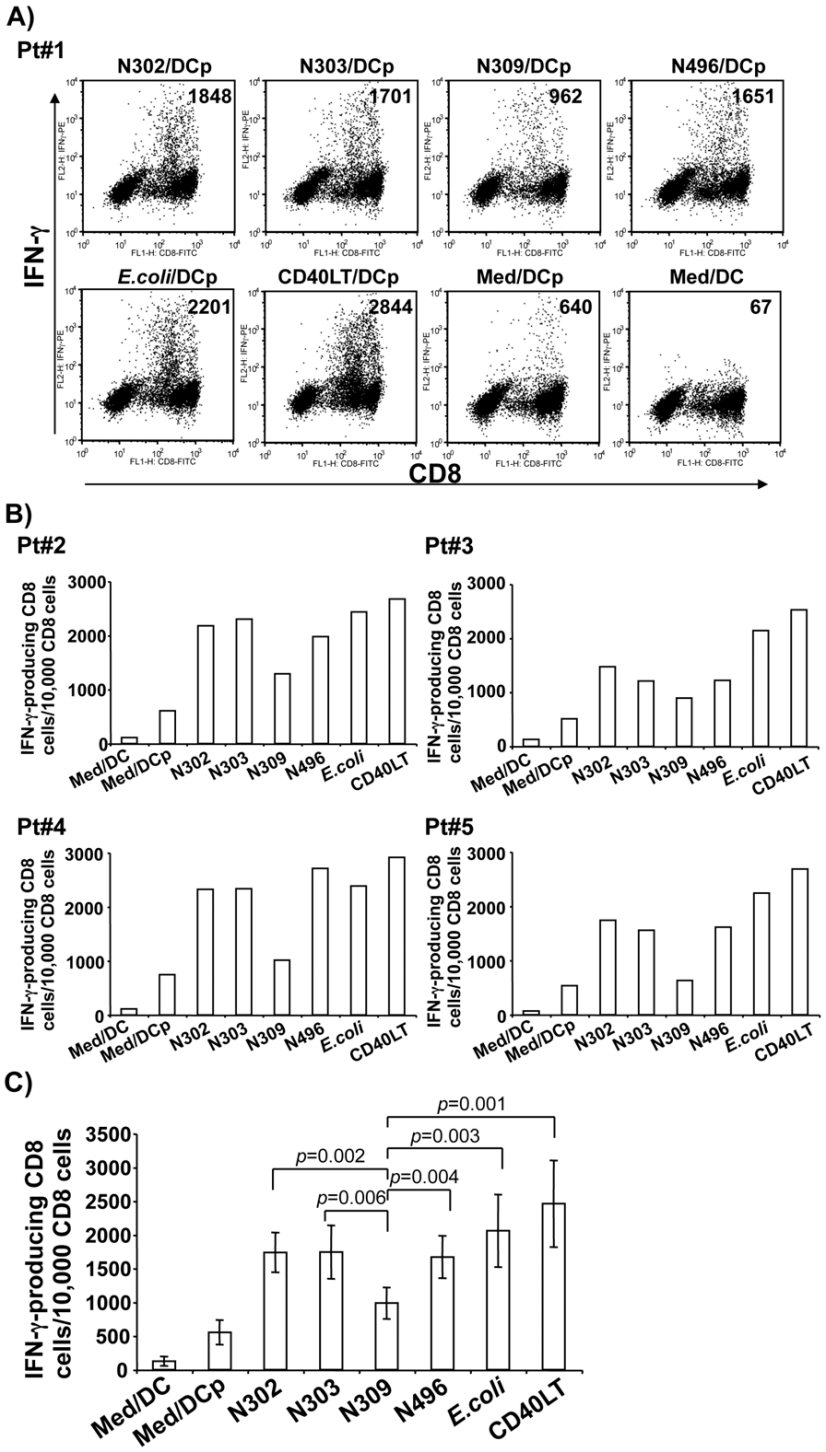
Effects of *N. gonorrhoeae* infection on HIV-1-specific CTL memory response. PBMCs from HIV-1-seropositive individuals were cocultured with HLA-restricted peptide-pulsed or nonpulsed autologous MDDCs that were previously infected for 72 h with *N. gonorrhoeae* N302, N303, N309, or N496, or *E.coli* DH5α, or CD40LT treatment at 1 µg/ml. On day 10 of coculture, HIV-1-specific CTL activity was assayed by intracellular staining and flow cytometric analysis of IFN-γ-producing CD8^+^ T cells. ***A)*** representative intracellular IFN-γ data obtained from Pt#1 of HIV-1 seropositive individual are shown. Cells were gated for CD3 and CD8 to enumerate IFN-γ-producing CD8^+^ T cells only. ***B)*** Summary data of intracellular IFN-γ production in CD8^+^ T cells from Pt#2 – Pt#5 are graphically depicted. ***C)*** Summary data of IFN-γ-producing CD8^+^ T cells from all patients tested are graphically depicted. The experiments from Pt#1 and Pt#2 were repeated with similar results. Statistical comparisons of data pooled from five participants were performed between Opa_CEA_-expressing N309 and other gonococci strains, *E.coli* DH5α, or CD40LT treatment: N309 vs N302, *p* < 0.05; N309 vs N303, *p* < 0.05; N309 vs N496, *p* < 0.05; N309 vs *E.coli*, *p* < 0.05; N309 vs CD40LT, *p* < 0.001; DC  =  DC not pulsed with peptides; DCp  =  DC pulsed with peptides; CD40LT  =  CD40 ligand trimer.

After determining that infection with the Opa_CEA_-expressing *N. gonorrhoeae* N309 impaired MDDC maturation and decreased MDDC-induced T cell proliferation, we sought to determine if Opa-CEACAM1 interactions would also alter an epitope-specific T cell memory response. To this end, we adapted our established *in vitro* CTL response assay in which peptide-pulsed MDDCs and T cells from the same individual are cocultured in the absence of exogenous cytokines to expand epitope-specific memory CTL responses [48], [49]. Five HIV-1 seropositive individuals with different rates of disease progression were studied: 2 long-term nonprogressors (LTNPs) (Pt#1 and Pt#4), 1 chronic progressor (Pt#3), and 2 recent seroconvertors (Pt#2 and #5) (Table 1). Immature MDDCs derived from these individuals were infected with the isogenic gonococcal strains or *E.coli* DH5α at an MOI of 10 for 3 days, and then pulsed with HLA-restricted peptides before co-culturing with autologous PBMCs. After 7–10 days of co-culture, CTL effector activity was assayed by measuring intracellular IFN-γ production after exposure to peptide-pulsed targets (autologous B lymphocyte cell lines or autologous T cell-depleted PBMCs). A representative experiment measuring HIV-1- specific CD8^+^ T cells producing IFN-γ by intracellular staining and flow cytometric analysis from Pt#1 is illustrated in Fig. 5A. A summary of the HIV-1-specific CD8^+^ T cells from Pt#2 to Pt#5 producing IFN-γ determined by flow cytometric analysis are illustrated in Fig. 5B. Summary pooled data from all five of HIV-1 seropositive participants are shown in Fig. 5C. Medium alone and CD40LT were included as negative and positive controls, respectively. As reported previously [49], [55], CD40LT-stimulated MDDCs strongly enhanced HIV-1-specific memory CTL responses when compared with medium-treated iMDDCs. In comparison with medium alone, MDDCs infected with the bacterial strains all enhanced HIV-1-specific memory CTL responses to a similar degree, with the exception that the Opa_CEA_-positive strain N309 induced a relatively weak response. Highlighting the dramatic effect of CEACAM1-specific Opa protein expression, the IFN-γ response elicited by the N309-infected MDDCs often reflected that with the peptide-pulsed but otherwise untreated DCs (986 ± 235, n = 5 vs 553 ± 181, n = 5)(Fig. 5A, 5B, 5C). Thus, infection of Opa_CEA_-positive gonococcal strain of N309 effectively suppresses the ability of DCs to stimulate HIV-1-specific memory CTL responses. This effect of Opa_CEA_-positive gonococcal strain of N309 on DC effector function corresponds with its inhibitory effect on CD83 expression and the allogeneic response, suggesting a potent ability of *N. gonorrhoeae* to prevent the DCs from eliciting a T cell response.

## Discussion

The success of *N. gonorrhoeae* as a colonizer of humans stems largely from its ability to persist in core groups of sexually active individuals within the population [70]. The gonococci have an incredible capacity to vary surface antigens, yet this characteristic is not sufficient to explain the absence of protection afforded against reinfection with the same serovar or, in some instances, an apparently identical strain [71]. The gonococcal Opa proteins that bind human CEACAM1 have previously been shown to cause a marked suppression in CD4^+^ T cell responses to a variety of activating stimuli [37]–[39], an effect that could presumably affect development and/or persistence of an adaptive response. More recent evidence suggests that bacterial binding to CEACAM1 may also suppress TLR2 (toll-like receptor 2)-mediated innate responses from infected epithelial cells [72], implying that Opa-CEACAM1 may facilitate both early colonization and longer term persistence within an infected individual.

Our current studies demonstrate that human iMDDCs are intrinsically better at binding *N. gonorrhoeae* than they are at adhering to *E. coli*, which was used as a prototypical Gram negative bacteria with its spectrum of innate immune agonists, throughout our studies. While gonococci that lack both the Opa and pilus adhesins can associate with the DCs, expression of one or more adhesins clearly facilitates this interaction. Despite this fact, iMDDCs exposed to either the *E. coli* or *N. gonorrhoeae* strains tended to show similar gross morphological changes and alteration in surface antigen expression typical of phenotypic maturation initiated in response to purified microbial-associated molecular pattern (MAMP)-containing molecules such as lipopolysaccharide (LPS) or the potent DC agonist CD40LT. In each case, the maturing DCs showed a similar pattern of upregulating their expression of the surface antigens, CD80, CD86, and HLA-DR, and down-regulated expression of CD1a. Strikingly, however, DCs that were infected with the Opa_CEA_-expressing strain of *N. gonorrhoeae* did not display any CD83, a commonly used marker of DC maturation and one that was apparent upon exposure to the other stimuli, including the isogenic gonococcal variants that lacked Opa_CEA_. The affect of Opa_CEA_ expression had functional consequences, since the DCs that matured in response to Opa_CEA_-expressing gonococci showed significant defects in their ability to elicit an allogeneic response against non-self leukocytes and in their ability to drive a peptide epitope-driven HIV-1-specific CD8+ T cell response against recall antigens.

A causal link for the absence of CD83 expression and defect in T cell stimulatory capacity of the Opa_CEA_-primed DCs is consistent with the recent demonstration that the specific siRNA-mediated knockdown of CD83 had a marked effect on their ability to stimulate T cell responses [69]. Indeed, even incomplete (∼60%) reduction in CD83 protein was sufficient to cause a significant reduction in T cell proliferation in an allogeneic mixed lymphocyte reaction, reflecting the effect seen with the CD83-negative but otherwise mature MDDCs arising following infection with the gonococci expressing Opa_CEA_. Curiously, several viruses, including herpes simplex virus type 1 (HSV-1) [73]–[75], human cytomegalovirus (HCMV) [76], HIV-1 [77] and vaccinia virus [78], have been shown to interfere with CD83 expression on the surface of infected DCs and thereby prevent DC-mediated activation and proliferation of T cells [79]. These viruses down-regulate CD83 expression through different mechanisms. For example, HCMV causes CD83 shedding from the surface of mature DCs [76] and the released soluble CD83 competes with membrane-associated CD83 to block DC-T-cell clustering [80], while HSV-1 strongly induces CD83 protein degradation in mature DCs via a process thought to be proteasome-mediated [74], [75]. HSV-1 infection also suppresses *de novo* expression of CD83 through degradation of cellular mRNA during DC maturation and blocks CD83 mRNA export from the nucleus into the cytoplasm [73], implying the importance of knocking out this protein for viral infection. Similar to the Opa_CEA_-expressing strain of *N. gonorrhoeae*, HSV-1 specifically diminishes CD83 without affecting other co-stimulatory molecules, leaving CD80 and CD86 on the surface of mature DCs [74], [75]. The molecular mechanisms by which these co-stimulatory molecules can be differentially regulated by DCs have not been studied. It has, however, been shown that CD83 expression is regulated at the posttranscriptional level by interaction of the shuttle protein HuR with the posttranscriptional regulatory RNA element (PRE) that is located in the coding region of the CD83 transcript [81]. HuR binds in a specific manner to the CD83 PRE region, and this interaction results in significant stabilization of the otherwise highly labile CD83 mRNAs [81]. As a consequence, HuR-CD83 mRNA binding ensures the timely and efficient nuclear export, and thereby protein expression, of CD83 transcripts [81]. When considered together with the *N. gonorrhoeae*-dependent effects shown herein, it is enticing to consider that CD83 downregulation must be a particularly effective mechanism for both viral and bacterial pathogens to disengage DCs and thereby prevent an effective immune response.

While *N. gonorrhoeae* can randomly phase vary expression of its eleven *Opa* genes on and off, Opa-negative variants are rarely found in clinical samples obtained from men [82]–[84] or women [85]; the only exception to this rule is during menstruation, when transparent (Opa-negative) colonies predominate [85]. The Opa-CEACAM1 dependent effects on DC and T cell effector function would presumably contribute to the absence of effective adaptive memory responses in response to gonorrhea, impacting CD4^+^ T cells’ role in the adaptive response and CD8^+^ T cell-mediated killing of *N. gonorrhoeae* infected cells. Either effect would allow gonococcal persistence in high-risk core groups of sexually active men and women. It may also contribute to the heightened transmission of HIV-1 apparent in co-infected individuals. Indeed, the relationship between these pathogens appears to occur at multiple levels since *in vitro* studies indicate that *N. gonorrhoeae* can dramatically enhance HIV-1 replication in humans CD4^+^ T cells [86] and DCs [56]. Moreover, gonococcal infection of genital epithelial cells promotes their release of pro-inflammatory cytokines that drive further HIV-1 expression [87]. When combined with an ability to suppress DC functions that are known to contribute to HIV-1 immunity, shown herein, and the direct suppression of T cell activation shown previously [37]–[39], this sets the stage for a dangerous liaison between two major sexually transmitted pathogens.
